# The Need for Earlier Diagnosis of Obstructed Hemivagina and Ipsilateral Renal Agenesis/Anomaly (OHVIRA) Syndrome in Case of Renal Agenesis in Girls—Case Report and Review of the Literature

**DOI:** 10.3390/jcm12237284

**Published:** 2023-11-24

**Authors:** Agnieszka Lecka-Ambroziak, Lidia Skobejko-Włodarska, Hanna Ruta

**Affiliations:** 1 Department of Endocrinology and Diabetology, The Children’s Memorial Health Institute, 04-730 Warsaw, Poland; 2 Endocrinology Outpatient Clinic, Institute of Mother and Child, 01-211 Warsaw, Poland; 3 Department of Urology, The Children’s Memorial Health Institute, 04-730 Warsaw, Poland; l.skobejko-wlodarska@ipczd.pl; 4 Gynaecology Outpatient Clinic, The Children’s Memorial Health Institute, 04-730 Warsaw, Poland; hania.ruta@gmail.com

**Keywords:** OHVIRA syndrome, renal agenesis, Müllerian ducts anomalies, endometriosis

## Abstract

Obstructed hemivagina and ipsilateral renal agenesis/anomaly (OHVIRA) syndrome is a very rare condition affecting girls. The time of diagnosis varies, from cases of prenatal diagnosis up to adulthood, including pregnancy or delivery. Most commonly, it is recognised during puberty and usually as an acute condition. We present a case report of an adolescent girl who underwent the treatment because of acute abdominal pain. The case is interesting due to a previous diagnosis of one-side renal agenesis. It appears to be useful to perform a diagnostic pelvic imaging at the time of diagnosis of renal agenesis or to plan to perform it at the beginning of puberty, to prevent the need for urgent surgery. This management may allow the planning of proper follow-up, minimising the risk of possible complications.

## 1. Introduction

Obstructed hemivagina and ipsilateral renal agenesis/anomaly (OHVIRA) syndrome, known also as Herlyn–Werner–Wunderlich (HWW) syndrome, is a very rare disorder related to obstructive Müllerian anomalies. The incidence of these disorders is estimated as 0.1–3.8% [1,2]. OHVIRA syndrome is a congenital anomaly of the genitourinary system, a result of the abnormal development of the Müllerian ducts around the eighth week of gestation, although the exact pathogenesis is not explained [1,2,3,4]. It is characterized by the presence of a double uterus (didelphys, bicornuate, or septate uterus, complete or partial), unilateral cervico-vaginal obstruction (obstructed hemivagina, communicant, non-communicant, or unilateral cervical atresia) and ipsilateral renal anomalies (renal agenesis/anomaly and/or other urinary tract anomalies) [1,2,3,4].

An obstructed hemivagina and uterus didelphys was first reported in 1922 [5]. The disease is also referred as HWW syndrome after the authors of the early case reports from 1971 to 1976 [2]. HHW historically refers to genital malformations and renal agenesis.

The clinical presentation varies in terms of time and severity of symptoms. Usually, OHVIRA syndrome is diagnosed during adolescence after menarche or in young adults. The main clinical symptoms include dysmenorrhea, usually progressive, menstrual irregularities, abdominal or pelvic pain or masses [2], and rarely urinary incontinence or urine retention [6,7,8,9]. However, in severe cases, previously undiagnosed OHVIRA syndrome may result in acute complications, such as pelvic infections, among them abscesses, pyosalpinx, and peritonitis, with potentially even septic shock [10].

There are also described cases of very early prenatal diagnosis [2,11,12], as well as delayed diagnosis during pregnancy or in labour [13,14,15,16]. In long-term observations, undiagnosed OHVIRA syndrome may lead to serious complications in adulthood as a result of retrograde flow with recurrent pelvic infections and endometriosis, with possible ovarian endometrioma [1,2,6], infertility, or miscarriage [2,17]. There are also other possible pregnancy complications that may directly influence a foetus or a newborn, such as abnormal foetal position, preterm delivery, or intrauterine growth restriction (IUGR) [18].

MRI provides details of uterine and vaginal morphology as well as allowing the diagnosis of different renal abnormalities [1,19,20,21]. There are no clear recommendations for the optimal time and method of surgical treatment for all patients. However, most published papers refer to the most minimally invasive option of vaginal septum resection [22,23,24,25]. A delay in proper diagnosis is not uncommon and has been reported by different authors [19,24].

We present a case report of an adolescent girl with acute symptoms of abdominal pain caused by an obstructed hemivagina in the course of undiagnosed OHVIRA syndrome with an earlier diagnosis of renal agenesis. We summarize the previous publications to review the different clinical presentations and recommendations in this rare disorder.

## 2. Case Report

**Figure 1:**
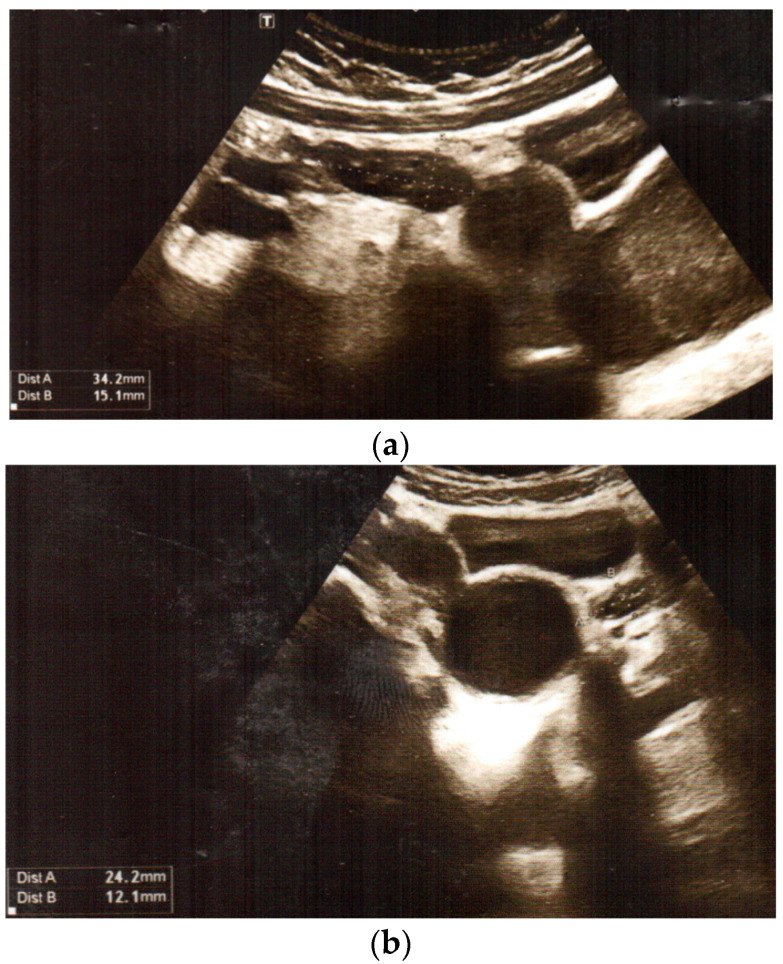

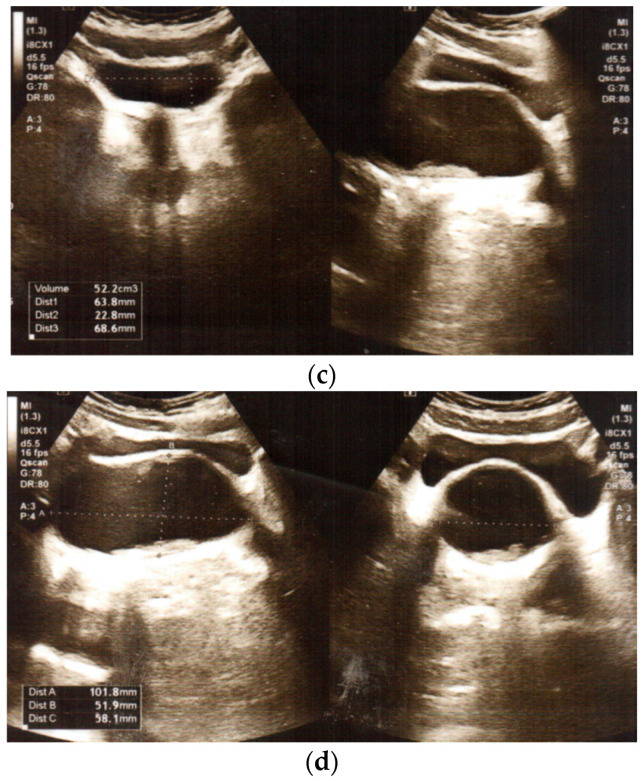
Transabdominal pelvic ultrasound at the time of diagnosis: (**a**,**b**) duplication of uterus: uterus with right-sided oval hypoechogenic structure, and normal ovaries (**c**,**d**) duplication of vagina, with haematocolpos on the right side.

We present the medical history of a 16.5-year-old girl who underwent urgent surgery at the age of 12 years. The girl was admitted to the Department of Urology due to complaints of severe abdominal pain on the sixth day of menstruation, with bladder pressure. There was no reaction to analgetic or spasmolytic medications. Transabdominal pelvic ultrasound examination revealed duplication of the uterus (uterus with right-sided oval hypoechogenic structure) and vagina, with a right vagina of 52 mm × 58 mm × 10 mm filled with blood (haematocolpos), normal ovaries (Figure 1). Abdominal ultrasound showed right renal agenesis with normal left kidney and ureter, and constricted bladder.

Previous medical history revealed menarche 5 months earlier with normal menstruation up to the current event. Abdominal ultrasound performed 2 years previously showed right renal agenesis with no further diagnostics. An urgent surgery was performed with vaginal septum excision up to the level of the double cervix, by the access from the left vagina.

A future follow-up had been planned. After surgery, the patient presented with regular, normal menses. She also received nephrological follow-up; the left kidney showed normal function.

At the age of 15.5 years, the girl presented at the Endocrinology Outpatient Clinic with mildly elevated TSH. The laboratory tests showed normal thyroid and adrenal function. Family history revealed only autoimmune thyroiditis in her older sister.

## 3. Literature Review

Due to rarity of the disorder, a limited number of analyses of patient series has been published so far. These are retrospective studies, mainly from one centre. Below, we summarise the relatively large series of patients with OHVIRA syndrome published in the last 25 years. The studies are varied with respect to the age of the patients, prepubertal and pubertal girls as well as adult women, main complaints, and anatomical variants. Different options of surgical treatment are presented together with possible complications of the syndrome itself and of the surgery. Moreover, available obstetric outcomes are reported. This summary provides a wider view of the clinical spectrum of the OHVIRA syndrome (Table 1).

In the first cited study, published in 1999 by Haddad et al., surgery options were analysed. Among laparoscopic procedures, vaginal septum excision was performed in most of the cases, with hemihysterectomy with ipsilateral hemicolpectomy just in 12% of the patients. Long-term results in 38 patients revealed dysmenorrhoea and abdominal pain resolved in 87% and 100%, respectively. The obstetric outcome seemed to be related to the type of uterine malformation [26]. An interesting publication from 2007 analysed patients from two centres, paediatric and gynaecologic, that had been assessed by one gynaecologist. Although most of them presented with renal agenesis, three had other ipsilateral renal anomalies, such as duplicated ureter and dysplastic or polycystic kidneys. The last two patients underwent nephrectomies in infancy. Regarding treatment, most patients again underwent single-stage vaginoplasty [27].

The second 2007 article discusses the importance of MRI diagnostics. Ultrasound examinations allowed the correct diagnosis of uterovaginal duplication, haematocolpos, or haematometrocolpos, and ipsilateral renal agenesis. However, MRI provided more detailed description of uterine morphology and the continuity with each vagina, obstructed and non-obstructed [28]. In contrast, in 2020, Zhang et al. concluded that MRI provides a comprehensive preoperative evaluation and identification of obstructive sites but not vaginal communications [37]. Ugurlucan et al. presented an interesting study with comparison the MRI measurements of the distance from the haematocolpos to the perineum, in patients who underwent single-stage vaginoplasty and hemihysterectomy. The conclusions confirm that the main treatment of OHVIRA syndrome is single-stage vaginoplasty, but hemihysterectomy may be necessary in cases of extreme proximal vaginal septum or for serious infectious complications [36]. It is also worth mentioning that MRI facilitates an overview the urological anatomy in detail before the surgery, as shown by Zhang et al. [38].

Regarding the studies assessing whether asymmetry exists in the OHVIRA syndrome, right-side anomalies were reported in 61% of patients in a paper published in 2007, which was comparable to previously published data. The authors presented a hypothesis that left–right side asymmetry may be induced before organogenesis [29]. However, a more recent systematic review revealed that such prevalence may not exist [43].

Recent publications show different anatomic variants of OHVIRA syndrome and their frequencies [31,43]. In a large series from the University of Milan, reported in 2013, most examined cases showed the classic anatomic variant; however, in the rest of the group (27.6%), rare variants were found, with septate uterus or cervical agenesis. These findings may be relevant for choosing a proper surgical treatment [31].

The clinical picture of OHVIRA syndrome differs regarding age group. In an analysis of paediatric patients published in 2008 by Capito et al., the diagnosis was suspected prenatally in three patients, with the presentation of dysplastic cystic kidney and a sonolucent pelvic mass. The authors summarise frequent misdiagnoses of OHVIRA syndrome during the paediatric period [30]. In another study from 2016, 23.3% of patients were diagnosed prenatally. The authors propose closer monitoring for urinary complications in prepubertal period, especially before the age of five years [34]. A novel study from 2019 presented adolescent patients in the post-menarche period, but not yet sexually active, who were managed by vaginoscopic incision of the oblique vaginal septum using a “No-touch” technique. The method is described as safe, minimally invasive, and effective [35]. In recently published paper by Zarfati and Lucchetti (2022), the girls were followed-up in the pre-menarche period and underwent surgery after menarche [40].

If undiagnosed, this disease may lead to recurrent spontaneous miscarriages or infertility in later life. In a 2013 paper from Peking Union Medical College Hospital, Tong et al. reported (with an update in 2015 by Zhu et al.) that the clinical presentation varied significantly between patients with complete and incomplete hemivaginal obstruction [32,44]. In the follow-up after vaginal septectomy, most of the women (84.8%) who wished to conceive became pregnant [32]. In a subsequent paper from the same centre, published in 2014, Wang et al. described analysis regarding the anatomical type of the syndrome. Types with perforate oblique vaginal septum or imperforate oblique vaginal septum and cervical fistula may not be diagnosed until reproductive age. There were 17 pregnancies reported in 15 women [33]. A 2021 publication described infertility as the leading symptom. During the follow-up after surgery in the seven patients that wished to conceive, five living infants were born [39]. The above results show that the surgical treatment may not only relieve the symptoms but also provide a positive obstetric outcome in the affected women.

This year has already brought two significant publications, one from Malaysia and one in a Chinese population. The first publication compares adolescent vs. adult groups. During adolescence, only two patients (11.1%) were asymptomatic. The authors reported that clinical presentation of acute abdominal pain is more common in the adolescent group [41]. In a second short report from 2023, Song et al. analysed the largest nationwide cohort of patients with OHVIRA syndrome, from 19 Chinese centres. The authors studied the frequency of extragenital malformations, such as scoliosis, abnormal cardiac development, or intestinal malrotation. The conclusions include possible recommendations for the evaluation of the skeletal system in the patients with OHVIRA syndrome [42].

## 4. Discussion

Among the many proposed classification systems for Müllerian ducts anomalies, one of the latest is the American Society for Reproductive Medicine Müllerian Anomalies Classification 2021 (ASRM MAC2021) [45]. It aimed to improve the American Fertility Society (AFS) Classification from 1988 that, although widely used, is mainly focused on uterine anomalies and does not recognise complex aberrations. In the new classification, anomalies are identified by descriptive terminology: Müllerian agenesis, cervical agenesis, unicornuate uterus, uterus didelphys, bicornuate uterus, septate uterus, longitudinal vaginal septum, transverse vaginal septum, and complex anomalies. There are also diagnostic procedures and treatment options included. The ASRM MAC2021 web application was developed to raise awareness of this rare anomalies.

Moreover, the diagnosis of OHVIRA syndrome is more common in recent years and it appears to be the most common obstructive Müllerian anomaly during the adolescence [46]. It has been shown that it may include more complicated genital anomalies, as well as different renal anomalies, not only renal agenesis but also kidney dysplasia or ureter anomalies [34,43,47]. In 2022, Feng et al. proposed a new classification system specifically for the manifestations of OHVIRA syndrome [3]. It is important to remember that prenatally diagnosed renal abnormalities are more common nowadays and in these cases in girls there is a need to plan a future follow-up, not only nephrological but also gynaecological. In rare cases, the disorder is revealed in women during pregnancy or at delivery and may cause serious complications at the time.

We reviewed the publications regarding a relatively large series of patients in a chronological order, to evaluate how the clinical picture and diagnostic and surgical options have been evolved. Interestingly, the most recent and the largest cohort reported this year, from Chinese centres, shows the possibility of association with other anomalies, so the future knowledge of the syndrome will be surely more detailed [42]. The authors of most of the papers conclude that there is a need for age-specific management and long-term follow-up, both pre- and post-surgery.

In the presented case, renal agenesis was diagnosed two years before the surgery for OHVIRA syndrome, at the age of 10 years, outside the hospital department. After excluding the first-line differential diagnosis of renal ectopy, gynaecological assessment with the pelvic ultrasound examination should have been performed or scheduled at that time. Therefore, the proper follow-up or treatment could have been planned, to avoid the necessity of urgent surgery.

In a case report published in 2011, renal agenesis was even diagnosed prenatally, but the clinical picture in adolescence was similar to that the presented patient [48]. The authors underline the necessity of a screening for congenital abnormalities of the reproductive tract in cases of diagnosed renal anomalies and vice versa.

In our opinion, the knowledge of OHVIRA syndrome should be disseminated between different clinical specialities. Although the disease is rare, the patients may be assessed due to their symptoms in different outpatient clinics or hospital departments, not only obstetrics and gynaecology, urology or surgery, but also nephrology, gastroenterology, endocrinology, or paediatrics. The diagnostics may also be difficult for radiologists because of the rarity of the syndrome. Apart from acute complications, the long-term risk of endometriosis or infertility should be considered.

## 5. Conclusions

It is worth considering OHVIRA syndrome as a differential diagnosis in female patients with renal agenesis or other renal and urinary tract anomalies. The early diagnosis of this rare disorder is essential to plan proper follow-up and choose the correct option for surgical treatment. Consequently, proper management may prevent the acute and long-term complications, with pelvic infections, endometriosis, infertility, and miscarriages among the most serious.

## Figures and Tables

**Table 1:** Summary of the literature review in a chronological order.

Year [Reference]	Authors	Years of Analysis	Patients Number (Age in Years, Range)	Main Results/Conclusions
1999 [26]	Haddad, B., Barranger, E., Paniel, B.J.	1970–1997	42 (mean 18, 11–30)	Endometriosis—37% Pregnancies—20 in 9 patients after vaginal septum excision (ipsilateral in 20%); 13 living children No pregnancies in 5 patients after hemihysterectomy and hemicolpectomy
2007 [27]	Smith, N.A., Laufer, M.R.	1992–2004	27 (median 14, 10–29)	Endometriosis—3 patients Misdiagnoses—5 patients Adenosis of vaginal septum—7 patients Postoperative vaginal stenosis—2 patients Rectovesical band—1 patient Urethrotomy due to a urethral diverticulum—1 patient
2007 [28]	Orazi, C. et al.	2000–2006	11 (11–15)	Misdiagnoses—2 patients MRI examination—more detailed description of uterine morphology and continuity with each vagina, obstructed and non-obstructed
2007 [29]	Vercellini, P. et al.	1991–2005	41	Asymmetry—right side anomalies in 61%
2008 [30]	Capito, C. et al.	1984–2007	32 (group 1, 8 patients: median 6 months, birth—6 years, group 2, 24 patients: 14, 11–17)	Misdiagnoses—9 out of 11 patients managed in emergency settings Delay in diagnosis after menarche of median 9 months (1–48)
2013 [31]	Fedele, L. et al.	1981–2011	87 (mean 20.7, 11–42)	Rare syndrome variants, with septate uterus or cervical agenesis—27.6%
2013 [32]	Tong, J., Zhu, L., Lang, J.	1995–2010	70 (mean age of the symptoms: Complete (20) vs. incomplete hemivaginal obstruction (50) 12.86 ± 1.84 vs. 20.68 ± 7.43)	Endometriosis—17.1% (25% vs. 14%, respectively) Pregnancies—52 in 28 patients of 32 who wished to conceive (ipsilateral in 36.5%)
2014 [33]	Wang, J. et al.	1985–2009	61	Analysis of symptoms in relation to anatomical type Misdiagnoses—11 patients Endometriosis—5 patients Postoperative vaginal stenosis after incomplete septum resection—5 patients Pregnancies—17 in 15 patients (ipsilateral in 52.9%)
2016 [34]	Han, J.H. et al.	2004–2015	43 (median 1.3 months)	Multicystic dysplastic kidney—65.1% Ectopic ureter insertion—62.8% Urinary tract infections, uncontrolled vaginal extension, urinary incontinence, abdominal pain—6 patients required surgery at median age 31.2 months (nephrectomy—5, vaginal septum resection—3) Impaired renal function—2 patients Recommendation of a closer monitoring for complications in prepubertal period
2019 [35]	Cheng, C. et al.	2009–2017	14 (10–19)	Vaginoscopic incision of the oblique vaginal septum with a “No-touch” technique (minimising underdeveloped vaginal wall disruption in the adolescent girls)
2020 [36]	Ugurlucan, F.G. et al.	2001–209	32 (mean 16.8 ± 6,4, menarche 12.8 ± 1.0)	MRI measurements of a distance from haematocolpos to perineum in 19 patients, compared with a type of surgery vaginoplasty (15) vs. hemihysterectomy (4): 33.9 ± 18.1 mm (10–79 mm) vs. 87.3 ± 11.0 mm (80–100), respectively (*p* = 0.009) Confirmation of a single-stage vaginoplasty as the main treatment
2020 [37]	Zhang H. et al.	2014–2019	40 (mean 20, 11–53)	MRI allows a comprehensive preoperative evaluation and identification of obstructive sites but not vaginal communications
2020 [38]	Zhang J. et al.	2009–2017	26 (mean 15.5, 10–31)	MRI provides a detailed overview of urological anatomy before the surgery
2021 [39]	Yi, S., Jiang, J.	2007–2019	17 (median age at surgery—23 years)	Infertility—the most common symptom Misdiagnoses—41.2% Endometriosis—1 patient Pregnancies—5 living infants in 7 patients that wished to conceive
2022 [40]	Zarfati, A., Lucchetti, M.C.	2009–2021	28 (mean 11.9, 25% before menarche)	Endometriosis—1 patient Impaired renal function—1 patient Reoperation—2 patients Follow-up in the pre-menarche period, surgery after menarche
2023 [41]	Lim, L.M. et al.	2013–2022	18 (group 1, 9 patients: 10–19, group 2, 9 patients: ≥20)	Misdiagnoses—4 patients Acute abdominal pain—more common in the adolescent group
2023 [42] Nationwide study Short report	Song, S., Chen, N., Zhu, L.	2015–2021	255	Scoliosis—14.5% (lumbar or sacral spina bifida in 7.1% of cases) Possible recommendations for additional evaluation of the skeletal system

## Data Availability

No new data were created or analyzed in this study. Data sharing is not applicable to this article.
